# Algorithmic Optimisation Method for Improving Use Case Points Estimation

**DOI:** 10.1371/journal.pone.0141887

**Published:** 2015-11-09

**Authors:** Radek Silhavy, Petr Silhavy, Zdenka Prokopova

**Affiliations:** Faculty of Applied Informatics, Tomas Bata University in Zlin, Zlin, Czech Republic; Nankai University, CHINA

## Abstract

This paper presents a new size estimation method that can be used to estimate size level for software engineering projects. The Algorithmic Optimisation Method is based on Use Case Points and on Multiple Least Square Regression. The method is derived into three phases. The first phase deals with calculation Use Case Points and correction coefficients values. Correction coefficients are obtained by using Multiple Least Square Regression. New project is estimated in the second and third phase. In the second phase Use Case Points parameters for new estimation are set up and in the third phase project estimation is performed. Final estimation is obtained by using newly developed estimation equation, which used two correction coefficients. The Algorithmic Optimisation Method performs approximately 43% better than the Use Case Points method, based on their magnitude of relative error score. All results were evaluated by standard approach: visual inspection, goodness of fit measure and statistical significance.

## Introduction

Software development effort estimation plays an important role in the software engineering field. There are several approaches under investigation. The first is method-based Functional Points Analysis (FPA), which has an official technical standard [1] in ISO standardisation. This method depends on the ability and experiences of an analyst, who is responsible for evaluating parameters. The system is descripted as set of functions, but the analyst must understand the system in great detail. A tendency of personal influence on the estimation can be described in the case of each individual analysis. Functional analysis considers a system as a black box. It is not important to describe the platform, the development method or any hardware used. The results of the calculation estimate only the effort of each individual system function or ability. The personal opinion of the analyst, which influences the estimation, makes this method unsuitable for comparing productivity in system or software development. However, if the same analyst prepares the calculation, the quality of the original estimation can be measured. If the results of the analysis in each development phase are compared and the error curve is flat, then the estimation is calculated accurately. This method is based on interactions between computer systems.

FPA considers external inputs or outputs. The boundary is the border between two systems from the user perspective. External inputs are processes that send data into the boundary. This input can be the control input or data input. The data may be used to maintain one or more internal logical files and can be either control information or business information. External outputs consider data from one or more internal logical files (ILFs) and external interface files. The input process does not update the ILFs, and the output side does not contain derived data.

An estimation technique for early stage estimation is called Use Case Points (UCP) method, which was developed by Gustav Karner [2]. His perspective addresses object-oriented design and is thus widely used. The method is based on use case models, which are commonly used as functional descriptions of proposed systems or software. A number of use case steps were initially involved in the estimation process. There have been several modifications of the original principles, such as use case size points [3], extended use case points [4], use case points modified [5], adapted use case points [6], and transaction or path analysis [7].

Karner’s basic method is based on assigning weights to clustered actors and use cases. Karner’s UCP method identifies three clusters–simple, average and complex. The sum of weighted actors creates a value called unadjusted actor weights (UAW), and in the same sense, the unadjusted use case weights (UUCW) value is calculated. Two coefficients, technical factors and environmental factors, are used to describe the project, related information and development team experience. Summing UAW and UUCW and then multiplying this value by the technical and environmental factor coefficients obtain the number of UCP. Finally, Karner [2] works with the productivity factor, i.e., the number of man-hours per point. This factor is one of the greatest issues with the methodology, as this final operation can change the estimate considerably.

Use case size points is investigated in [3]. The authors emphasise the internal structure of the use case scenario in their method. The primary actors take roles and classifications based on the adjustment factor. Fuzzy sets are used for the estimation.

Several authors have presented improvements to Karner’s method based on identifying transactions instead of steps in use cases. In [7], the number of stimuli is equal to the number of transactions. A stimulus is an activity of an actor in a use case. The authors of [1] and [7] improve transactions by calculating paths. The final complexity of the transaction is based on the number of binary or multiple conditions used in the scenarios.

The second modification described here is prepared by Wang [4] and is called extended UCP. This approach employs fuzzy sets and a Bayesian belief network, which is used to set unadjusted UCP. The result of this approach is a probabilistic effort estimation model.

Diev [5] notes that if the actors and use cases are precisely defined, then unadjusted UCP can be multiplied by technical factors. Technical factors are used to establish a coefficient of base system complexity. According to [5], the effort of further activities must be added. These further activities are supportive tasks, such as configuration management or testing.

The next interpretation is called adapted UCP [6]. UCP are adapted to incremental development for large-scale projects. In the first increment, all actors are classified as average and all use cases as complex. The authors also propose the decomposition of use cases to smaller ones, which are classified into the typical three categories.

Use case-based methods of estimation have certain well-known issues [8]. Use cases are written in natural language, and there is no rigorous approach to allow for a comparison of the use case quality or fragmentation. Therefore, the number of steps may vary, which affects the estimation accuracy. In a single individual use case, there can be more than one scenario, which can influence the accuracy of the estimation. Thus, the use case model is critical for system functional or behavioural modelling, but for the purpose of estimation, use cases can be used only if the estimation approach can be adjusted or calibrated for individual work conditions and writing style. Here, we propose an algorithm of calibrating the model by setting the productivity factor according to historical data.

## Methods

### UCP Method Description

In the UCP method [1, 2, 4, 9], actors and use cases are classified as simple, average or complex [2]. A simple actor typically represents an application programming interface (API) or, more generally, a non-physical system user. A system interconnected by network protocol is classified as average, and finally, physical users interacting with the system through a graphical user interface are complex actors. These factors are summarised in Table 1.

**Table 1:** Actor Classification and Actor Weighting Factor.

Actor Classification (AC)	Weighting Factor (WFa)
Simple	1
Average	2
Complex	3

The total unadjusted actor weights (TUAW) are calculated according to the following formula:

TUAW=∑AC×WFa (1)

Use cases are classified in a similar manner (see Table 2). The complexity of use cases is based on the number of steps, also called a transaction. However, a transaction typically refers to a path of activity, not a simple step in a structured scenario. Therefore, the term “step” is used here. The step counting process is not clear from the inline view. The original method counts steps in a primary scenario and in alternative scenarios. If the use case is extended (or included) by another use case, these steps are not counted: these use cases are counted as separate scenarios.

**Table 2:** Use Case Classification and Weighting Factor.

Use Case Classification (UCC)	Number of Steps	Weighting Factor (WFb)
Simple	(0,4)	5
Average	<4,7>	10
Complex	(7, ∞)	15

The unadjusted use case weights (UUCW) are calculated according to the following formula:

UUCW=∑UCC×WFb (2)

Technical factors (first correction value) are then applied to the unadjusted UCP (UUCP). The technical complexity is already known in the FPA method. The second correction value is based on environmental factors. This factor describes non-functional requirements. Table 3 presents the technical factors, and Table 4 presents the environmental factors, as they are known in UCP.

**Table 3:** Technical Factors.

Factor ID	Description	Weight (WFc)	Significance (SIa)
T1	Distributed System	2	<0,5>
T2	Response adjectives	2	<0,5>
T3	End-User Efficiency	1	<0,5>
T4	Complex Processing	1	<0,5>
T5	Reusable Code	1	<0,5>
T6	Easy to Install	0.5	<0,5>
T7	Ease to Use	0.5	<0,5>
T8	Portable	2	<0,5>
T9	Easy to Change	1	<0,5>
T10	Concurrent	1	<0,5>
T11	Security Feature	1	<0,5>
T12	Access for Third Parties	1	<0,5>
T13	Special Training Required	1	<0,5>

**Table 4:** Environmental Factors.

Factor ID	Description	Weight (WFd)	Significance (SIb)
E1	Familiar with RUP	1.5	<0,5>
E2	Application Experience	0.5	<0,5>
E3	Object-oriented Experience	1	<0,5>
E4	Lead Analyst Capability	0.5	<0,5>
E5	Motivation	1	<0,5>
E6	Stable Requirements	2	<0,5>
E7	Part-Time Workers	-1	<0,5>
E8	Difficult Programming Language	2	<0,5>

Factors T1-T13 and E1-E8 have fixed weights. Moreover, for each factor, the significance can be set in the interval from 0 to 5, where 0 indicates no impact, 3 indicates an average impact, and 5 indicates a strong impact. The technical complexity factor (TCF), that is, the first correction coefficient, can be calculated according to the following formula [7]:

$$TCF\;=\;0.6+\left(0.01\;\times \sum_{T13}^{T1}\;WFc\;\times \;SIa\right)$$ (3)

The second correction coefficient is called the environmental complexity factor (ECF). The formula for calculation is similar to the above:

$$ECF\;=\;1.4+\left(-0.03\;\times \sum_{E8}^{E1}\;WFd\;\times \;SIb\right)$$ (4)

The final result of the estimation is called adjusted UCP (AUCP) and represents the project (system or software) size in points. The unadjusted value of points from actors and points from use cases are summarised, and then, the TCF and ECF are applied. The following formula is used [1, 2, 4, 9]:

UCP=(TUAW+UUCW)×TCF×ECF (5)

The UCP value represents a system size, but a man-hour value is used to measure the work effort. Therefore, UCP is typically multiplied by 20 (according to [2]) or, more generally, by 15–30 man-hours per point. This value is a productivity factor of the development team.

Transactions in a use case scenario have been investigated as a method of tuning the traditional UCP concepts. Robiolo et al. and Ochodek et al. [7, 10] introduce transaction-based methods. According to [7], a transaction is a part of a use case scenario that represents a stimulus. As an example, a scenario can be reduced to a set of pairs. If transactions are used, then the UUCW is different from that in an example with steps. Each use case scenario typically has more steps than transactions.

### Algorithmic Optimisation Method Design

The proposed Algorithmic Optimisation Method (AOM) design is motivated by investigating the possibility of solving issues with the standard estimation process, which are described in the UCP method description section. Therefore, we have applied an optimisation method based on increasing the correction coefficients accuracy by multiple least squares regression [11].

Furthermore, we simplify the estimation process itself and make it less sensitive to personal ability and experiences. New approach indeed brings straightforward approach, which is based on historical data. This principal idea allows minimalizing an error as integral part of estimation process.

The AOM is based on a widely used version of the UCP method, which was previously described in the text.

**Fig 1:**
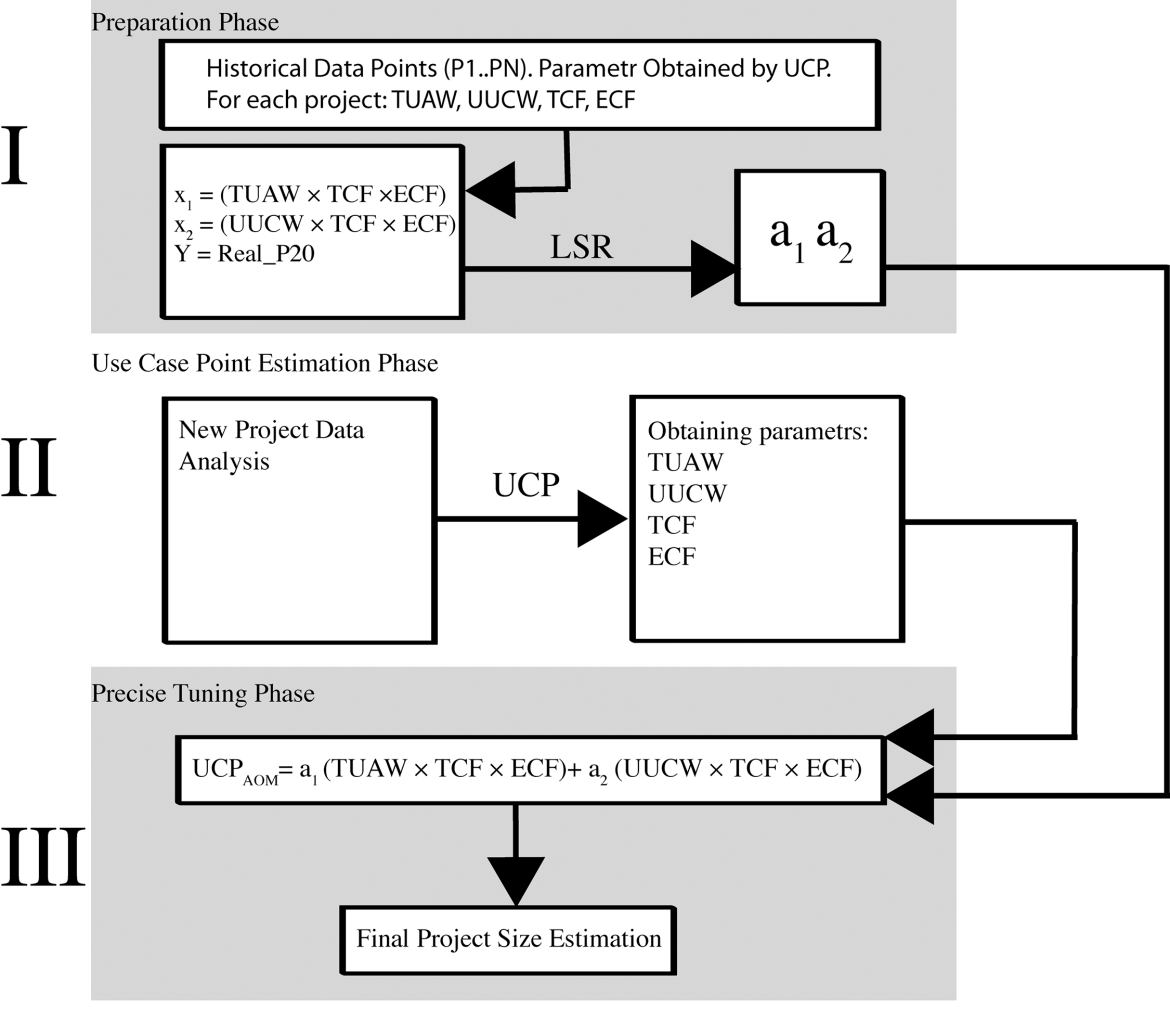
Algorithmic Optimisation Method Schema.

AOM brings tree-phases design, as can be seen in Fig 1. The Preparation Phase (Phase I) is used for calculation of the (*a*_1_, *a*_2_) correction coefficients. Least Squares Regression (LSR), or, more precisely, multiple linear regression is used for obtaining these coefficients (*a*_1_, *a*_2_). Following formula is applying for calculation:

$$\left(\begin{array}{c} y_{\begin{array}{c} 1\\ \vdots \end{array}}\\ y_{n} \end{array}\right)=\;\left(\begin{array}{cc} x_{\begin{array}{c} 11\\ \vdots \end{array}} & x_{\begin{array}{c} 12\\ \vdots \end{array}}\\ x_{n1} & x_{n2} \end{array}\right)\;\times \;\left(\begin{array}{c} a_{1}\\ a_{2} \end{array}\right)\Rightarrow \left(\begin{array}{c} a_{1}\\ a_{2} \end{array}\right)=\;{\left(X^{T}\;\times \;X\right)}^{-1}\;\times \;\left(X^{T}\;\times \;Y\right)$$ (6)

Where *x*_*i*1_ and *x*_*i*2_, *i* = 1…n, are as follows:

$$x_{i1}\;=\;\left(TUAW_{i}\;\times \;TCF_{i}\;\times \;ECF_{i}\right)$$ (7)

$$x_{i2}\;=\;\left(UUCW_{i}\;\times \;TCF_{i}\;\times \;ECF_{i}\right)$$ (8)

And *y*_*i*_ represents real size (Real_P20 values), which is real size of software projects form historical dataset (see Table 5). Therefore values of *a*_1_, *a*_2_ can be different for each individual dataset or generally for each project pool and developers. The values *a*_1_, *a*_2_ are not generally valid and their numerical values cannot be applied to other dataset or project. This method can be used only, if the historical data points exist. It is inevitable for correction functioning of AOM to perform Preparation Phase again, when historical dataset expands. New data points can be added to historical dataset only, if there is new finished project and its real size is known.

**Table 5:** Raw Project Data [9, 12].

Project No.	TUAW	UUCW	TCF	ECF	Est_P	Real_P20
1	12	375	0,92	0,78	277,7112	151,85
2	10	85	0,75	0,81	57,7125	95,85
3	6	115	0,9	1,05	114,345	58,65
4	9	205	0,85	0,89	161,891	37,1
5	12	160	0,82	0,79	111,4216	30,7
6	9	115	0,85	0,88	92,752	24,6
7	6	85	0,78	0,51	36,1998	13,85
8	14	515	0,94	1,02	507,2052	179,65
9	12	185	1,03	0,8	162,328	84,05
10	12	290	0,71	0,73	156,5266	67,2
11	9	150	1,05	0,95	158,6025	61
12	12	135	0,78	0,79	90,5814	36
13	4	60	0,96	0,96	58,9824	25,7
14	15	160	0,9	0,91	143,325	19,85
15	15	355	1,125	0,77	320,5125	184,2
16	18	145	1,08	0,77	135,5508	99
17	12	325	1,095	0,95	350,56425	197,5
18	6	90	1,02	1,085	106,2432	96,25
19	9	125	1,025	0,98	134,603	108,75
20	9	120	1,118	0,995	143,50089	111,3
21	12	200	1	0,92	195,04	132
22	9	175	0,95	0,92	160,816	128,4
23	12	245	0,89	1,19	272,1887	152,1
24	6	140	0,965	0,755	106,37195	84,8
25	7	180	0,81	0,84	127,2348	183,5
26	7	155	0,9	0,94	137,052	143
27	5	210	0,72	0,67	103,716	137
28	6	340	0,8	0,74	204,832	168

Secondly the basic UCP parameters are calculated. Eqs (1–4) are used for obtaining of TUAW, UUCW, TCF and ECF parameters.

Adopting the part of UCP estimation methodology supports consistency early processes of estimation. These parameters can be obtained from various CASE tools.

Thirdly, Precise Tuning Phase is performing. *UCP*_*AOM*_ value represents final estimated project size, which should be used for project planning and later on for effort estimation. *UCP*_*AOM*_ is obtained according Eq (9).

$$UCP_{AOM}=\;a_{1}\left(TUAW\;\times \;TCF\;\times \;ECF\right)+\;a_{2}\left(UUCW\;\times \;TCF\;\times \;ECF\right)$$ (9)

### Experiment planning

The experiment in our study deals with evaluating AOM principles, which may significantly improve prediction model based on Use Case Points.

In order to validate effectiveness of AOM an empirical validation was conducted. The first model, which was evaluated, is a Use Case Points (UCP). UCP was used as simple base model for setting basic performance level.

The second model, which may be called, advanced is AOM. Finally, the accuracy of prediction of two presented models was compared.

Let assume, that the prediction error of AOM will be significantly lower, that error of UCP method and optimal values of correction coefficients *a*_1_, *a*_2_, will be set.

Our task is not to offer generally valid coefficients. This paper will demonstrate that AOM is applicable to several projects pool.

In order to decide whether the model is more capable for prediction, a statistical hypothesis was tested:

H_0_: UCP = AOM, There is no difference in the capability of prediction between UCP and AOM models. No difference in estimation errors.

Alternative hypothesis:

H_1_: UCP ≠ AOM, there is difference in prediction capability between UCP and AOM models. There is a difference in estimation errors.

This paper compares the accuracy of the AOM model with that of the UCP model using t-test. The t-test is used as a test of the null hypothesis that the means of two normally distributed populations are equal. The t-test will be used for evaluation of MRE.

### Adopted Metrics for Performance Evaluation

Magnitude of Relative Error (MRE), Mean Magnitude of Relative Error (MMRE) and Percentage of Prediction (PRED(0.25)) is accepted as standard evaluation in size prediction and effort estimation techniques. In this paper are used this methods for purpose of future cross comparison to other approaches.

We also adopted Total Sum of Squares (TSS), which as metric for evaluation of the prediction models and median of errors. Following equations are used for obtaining this performance metrics:

$$\text{MRE}\;=\;\frac{\left|PredictedSize-Real\_P20\right|}{Real\_P20}$$ (10)

$$\text{MMRE}\;=\;\frac{\sum_{i}^{N}\;MRE}{N}$$ (11)

$$\text{PRED}\left(0.25\right)\;=\frac{A}{N}$$ (12)

$$\text{TSS}=\;\sum_{i=1}^{n}\;{\left(Real\_P20-PredictedSize\right)}^{2}$$ (13)

*PredictedSize* value stands for values of predicted project size. This value is a value of UCP or AOM. A “N” in Eqs (11) and (12) stands for number of observed projects. Next metric is a PRED(0.25). An “A” in Eq (12) stands for number of projects, which have MRE<0.25.

Goal for results is to keep MMRE and TSS minimalized and PRED(0.25) maximized. For purpose of our evaluation the most important criterion is minimal value of TSS, because it means that LSR algorithm achieved optimal performance.

## Results and Discussion

In this paper the UCP method [2] and our proposed method, called the Algorithmic Optimisation Method (AOM) were compared.

Table 5 summarises the raw project data. The TUAW, UUCW, TCF and ECF are known from UCP. Est_P is an estimation (in points) according UCP method (see Eqs (1–5)) a Real_P20 is a value, which represents real project size, which were measured in points, when project were finished.

The results were evaluated against three datasets, which take role as simulated software project pools. Each testing dataset were constructed as random selection of 10 projects for Phase I (see Table 6) from raw project data (see Table 5). These datasets takes role as historical dataset. For the evaluation we have used rest of dataset. Our evaluation will at α = 0.05 significance level.

**Table 6:** Preparation Phase Datasets Characteristics.

Test Case	Historical Data Points	Real-P20 (Min)	Real-P20 (Max)	Mean	Standard Deviation
Test1	P23,P6,P4,P26,P18,P3,P8,P16,P17,P2	24,6	197,5	108,37	55,76
Test2	P20,P1,P8,P2,P3,P24,P12,P9,P27,P18	36	179,65	103,54	40,9
Test3	P19,P1,P24,P27,P20,P22,P2,P11,P9,P5	30,7	151,85	99,37	34,48

Results of evaluation are based on rest of the dataset. It means that for evaluation were used:

Test1: P1, P5, P7, P9, P10, P11, P12, P13, P14, P15, P19, P20, P21, P22, P24, P25, P27, P28

Test2: P4, P5, P6, P7, P10, P11, P13, P14, P15, P16, P17, P19, P21, P22, P23, P25, P26, P28

Test3: P3, P4, P6, P7, P8, P10, P12, P13, P14, P15, P16, P17, P18, P21, P23, P25, P26, P28

Projects, which were used during a Preparation Phase, were omitted. The obtained results are presented in Table 7. Real_P20 were obtained from Table 5 and estimated size was calculated according Eq (9) for AOM or according Eq (5) for UCP.

**Table 7:** Comparison of the Estimation Methods Performance.

	Test1	Test2	Test3
MMRE-UCP	1,17	1,39	0,69
MMRE-AOM	0,69	0,83	0,69
PRED(0.25)-UCP	0,17	0,17	0,17
PRED(0.25)-AOM	0,39	0,34	0,34
TSS-UCP	97229,35	129109,46	224825,52
TSS-AOM	52041,24	51708,79	50679,76

**Fig 2:**
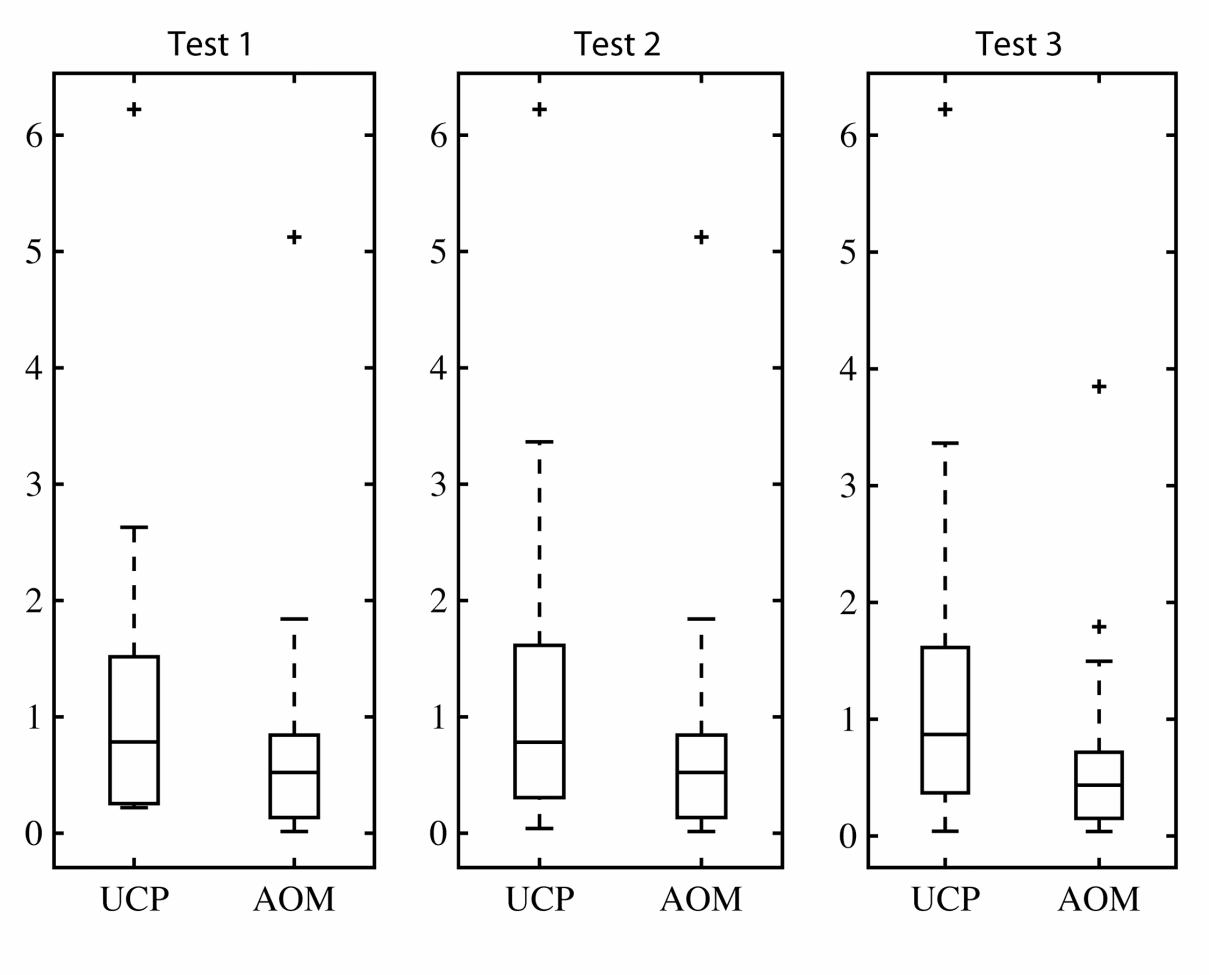
Box Plot on MRE Results obtained from testing datasets.

Fig 2 shows the Magnitude of Relative Error of the AOM for the Test1-3 datasets and compares them to UCP performance on same datasets. Table 7 and Fig 2 illustrate that AOM produces significantly better estimates than UCP. The mean MRE is 0.74 in the case of AOM, which is more than 43% better then result obtained UCP method. According to PRED(0.25) AOM is more then twice better.

**Fig 3:**
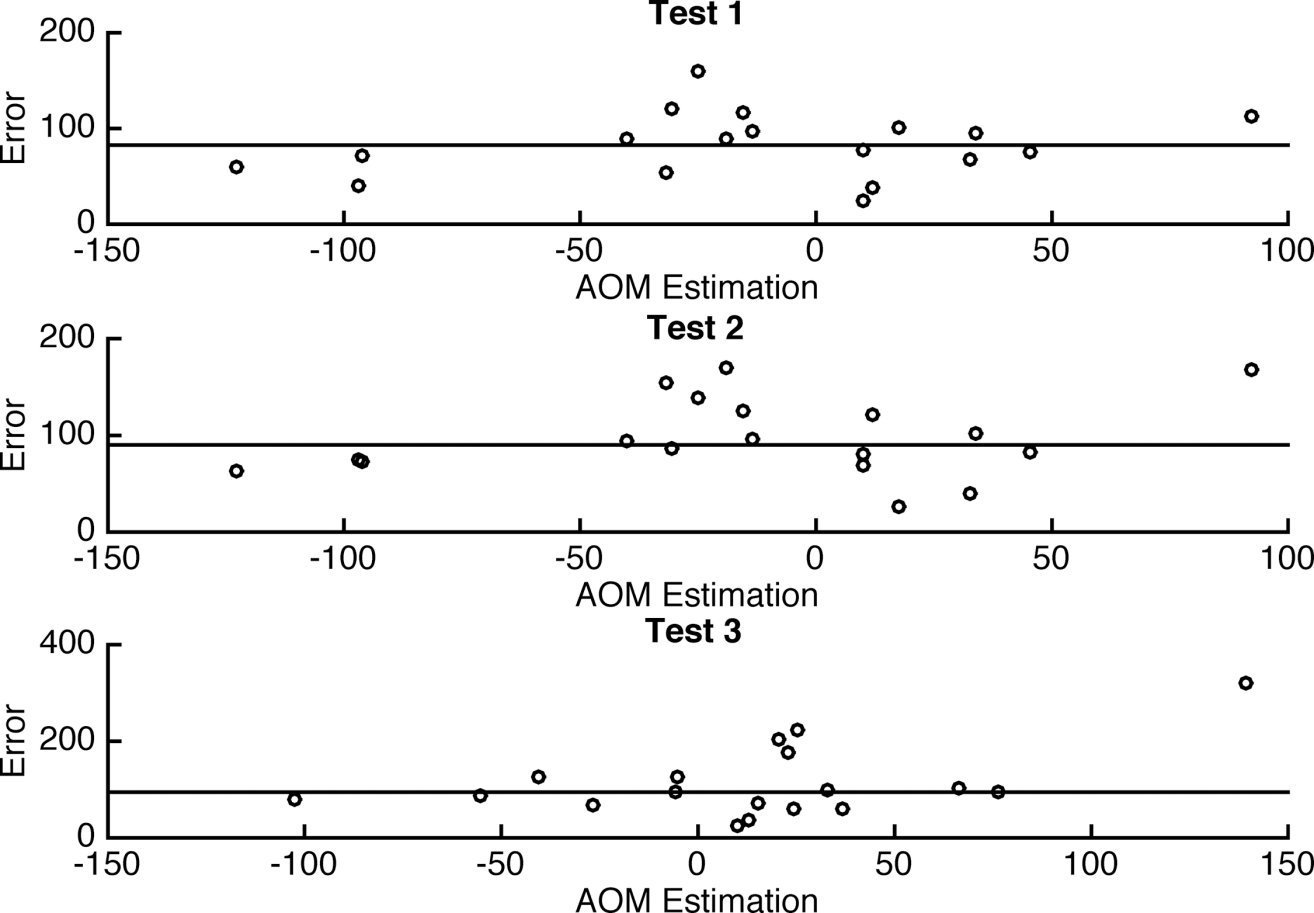
Error and AOM (PredictectSize) Correlation.

**Fig 4:**
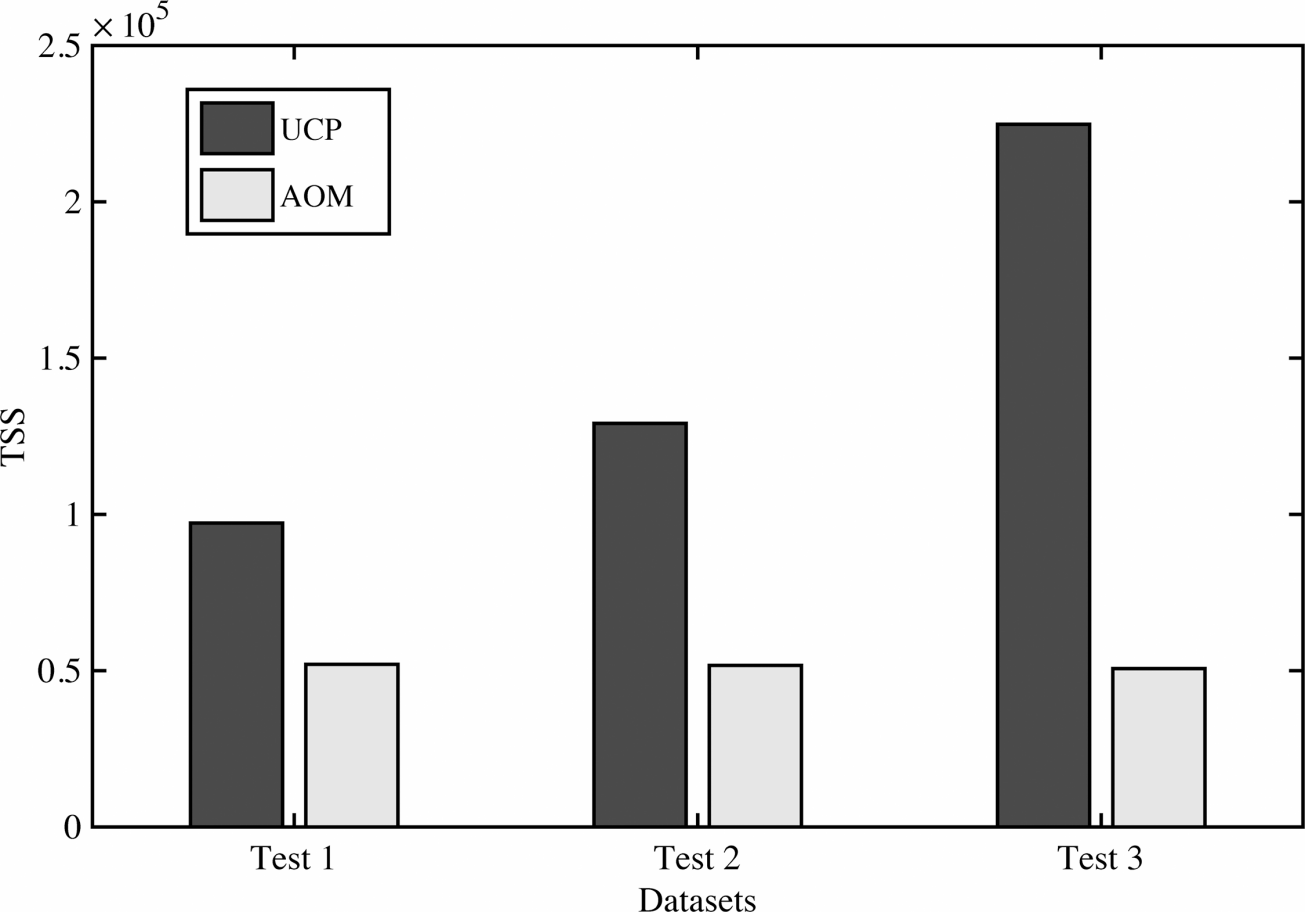
Total Sum of Squares.

Fig 3 describes a low correlation between Error and AOM predicted Size. The AOM performs consistently better as can be seen from Fig 2 (MRE distribution) and from Fig 4, which shows a Total Sum of Squares.

### Results Interpretation

Results, which are presented, in previous section show, that AOM model can produce significantly better estimations. AOM produce more reliable and more consistent results. The result of the t-test for MRE is given in Table 8. All datasets (Test1, Test 2 and Test3) were tested independently.

**Table 8:** Results of t-test for MRE.

	Test 1	Test 2	Test 3
SD	0.5992	0.6373	0.6685
Degree of freedom	17	17	17
t value	-3.3393	-3.7059	-4.3011
p value	0.0039	0.0018	0,00048385
h value	1	1	1

Results of t-test for MRE as a computational results (MRE, MMRE, PRED and TSS) show, that AOM performs significantly better. MRE was tested at significance level 0.05 and null hypothesis can be rejected. Performance of AOM and UCP is not equal.

### Validity Treats

There is an issue connecting with size of dataset. Use Case points dataset is not broadly available and therefore all studies are perform on relatively small datasets. We have used 28 data points, which were divided into two parts randomly. Three (Test 1, Test 2 and Test 3) dataset were created.

Our data points represent small and medium projects and for this should be a limitation. Therefore further investigation based on broad project pool and for bigger projects will be needed. Whereas it becomes apparent, that project size and project size variance is important parameter for model construction.

The main aim of this paper was to show that AOM approach could lead to more precise and consistent prediction than UCP. UCP is used as valuable initial estimation, from which some parameters are used for AOM estimation.

### Case Study

The following case study will illustrate real-life situation of using AOM. Case Study is descripted in phases according Fig 1.

#### Preparation Phase

Lets assume that there are 10 projects in historical dataset. Table 9 illustrates historical project data.

**Table 9:** Case Study Historical Data Points.

Project No.	TUAW	UUCW	TCF	ECF	Real_P20
P1	12	375	0,92	0,78	151,85
P2	10	85	0,75	0,81	95,85
P3	12	160	0,82	0,79	30,7
P4	9	115	0,85	0,88	24,6
P5	12	185	1,03	0,8	84,05
P6	9	125	1,025	0,98	108,75
P7	9	120	1,118	0,995	111,3
P8	9	175	0,95	0,92	128,4
P9	6	140	0,965	0,755	84,8
P10	5	210	0,72	0,67	137

These data points are used for setting *x*_1_ and *x*_2_ as is declared in Eqs (7) and (8). Real_P20 represents know value of real project effort.

Next step is to obtain (*a*_1_, *a*_2_) values. These are calculated by using multiple linear regressions. Values are as follows: *a*_1_ = 0.96737, *a*_2_ = 0.64416.

#### Use Case Points Estimation Phase

Values of TUAW, UUCW, TCF and ECF for the project, which is estimated, are obtained by UCP Eqs (1–4). Parameters values are: TUAW = 12, UUCW = 245, TCF = 0,89, ECF = 1,19.

#### Precise Tuning Phase

Finally project size estimation can be obtained by using Eq (9). For our project Equation is *UCP*_*AOM*_ = 0,96737 × (12 × 0,89 × 1,19) + 0.64416 × (245 × 0,89 × 1,19). Therefore, new project estimation is 179 use case points.

## Conclusions

In this article, we presented a new estimation approach based on a three-phase algorithm, called AOM. In the first phase, we applied a calculation based on UCP to obtain set of parameters (TUAW, UUCW, TCF, ECF) for each of historical project. Next we have obtain a correction values (*a*_1_, *a*_2_). These values were obtained via least squares regression. This approach employs historical project data to refine the estimation. By applying the least squares regression approach, the algorithm penalises errors in the estimation caused by human factors and company practice.

The AOM is significantly better than UCP estimation methods, with a mean MRE that is 43% better than that obtained with UCP.

Results of AOM are better and according Fig 4, results are significantly more consistent than those obtain by UCP.

The AOM represents an estimation workflow and describes approach how a size of software project could be estimated. AOM employs correction values (*a*_1_, *a*_2_), which are making a model less sensitive to introduction a personal errors.

As results clearly shows AOM produce consistent predictions. Ability of adaptation to individual project characteristics is improvement too.

A comparison drawn with regard to a number of descriptive characteristics has shown that datasets can influence the quality of the estimation. Therefore, in our future research, we will focus on dataset expansion and clustering projects into homogenised segments, which should improve the accuracy of the estimation.
